# Electroencephalography-Based Machine Learning for Biomarker Detection in Dyslexia and Autism Spectrum Disorder: A Comparative Review of Models, Features, and Diagnostic Utility

**DOI:** 10.3390/diagnostics15182388

**Published:** 2025-09-19

**Authors:** Günet Eroğlu

**Affiliations:** Computer Engineering Department, Engineering and Nature Faculty, Bahçeşehir University, Istanbul 34000, Turkey; gunet.eroglu@healthmobilesoftware.com

**Keywords:** dyslexia, autism spectrum disorder, machine learning, artificial intelligence, EEG biomarkers, synaptic connectivity

## Abstract

To uncover neurobiological indicators related to autism spectrum disorders and developmental dyslexia, this article gives a full overview of the most recent advances in machine learning and deep learning methods based on electroencephalography. We look into methodological pipelines that include signal gathering, preprocessing, feature engineering, model selection, and interpretability procedures. We based these pipelines on 15 peer-reviewed research papers published between 2013 and 2025. Most of the research employed the 10–20 system for resting-state EEG and followed MATLAB, MNE-Python, or EEGLAB guidelines for preprocessing. The feature sets included spectral power, functional connectivity, task-evoked potentials, and entropy measures. People used many standard ML methods, such as support vector machines and random forests, as well as more advanced models, like deep neural networks and transformer-based architectures. Several studies found that both dyslexic and ASD groups did well at classifying, with accuracy scores between 82% and 99.2%. The new models could be used in therapeutic settings, but there are still problems with how easy they are to understand and how well they apply to a wide range of situations. This is especially true for ASD because its spectrum is so varied.

## 1. Introduction

### 1.1. Defining Dyslexia and Autism Spectrum Disorder (ASD)

Dyslexia and ASD are two frequent but different types of neurodevelopmental disorders that affect learning, behavior, thought, and social skills in distinct ways through separate neural pathways. Dyslexia describes a group of conditions with a variety of symptoms, all resulting in difficulty reading and writing, even when adequate training has been given and intelligence levels are at or above the average. People with ASD have difficulty communicating with others and exhibit restricted, repetitive patterns of behavior, interests, or activities [1]. Both conditions are present in early life and have a great range of clinical severity.

### 1.2. Genetic and Environmental Contributions

Numerous genetic and environmental factors interact to cause ASD and dyslexia. Genetic loci like KIAA0319 and DCDC2, which are essential for synapse function and neuronal migration, have been found through genome-wide association studies [2]. These genes interact dynamically with environmental exposures rather than acting independently. For example, altered neurodevelopmental trajectories have been linked to prenatal factors such as vitamin D deficiency and maternal immune activation [3]. Therefore, environmental factors and genetic predispositions work together to determine whether the developing brain reacts maladaptively or adaptively during critical stages of growth, rather than acting independently.

### 1.3. Neuroimmune Dysregulation and Synaptic Pruning

More and more evidence has shown that neuroimmune processes play a significant role in synaptic pruning during the early stages of brain network formation. During early development, microglia, which are immune cells in the brain, facilitate the elimination of weak or unnecessary synaptic connections [4,5]. Disturbances in this process, whether excessive or inadequate, can alter the organization of neural circuits. While the precise role of synaptic pruning remains unclear, new research data suggests a potential link between dyslexia and changes in functional connectivity. Long-range fronto-temporal and parietal circuits, in particular, cannot sustain their phonological processing tasks under varying circumstances [6,7]. Individuals in different stages of growth demonstrate differences in these effects. However, both local hypersynchrony and abnormal large-scale network dynamics in ASD have been linked to aberrant microglia activation and altered immune signaling [4].

### 1.4. The Significance of Machine Learning (ML) and Electroencephalography (EEG) as a Window into Neurodevelopment

**Figure 1:**
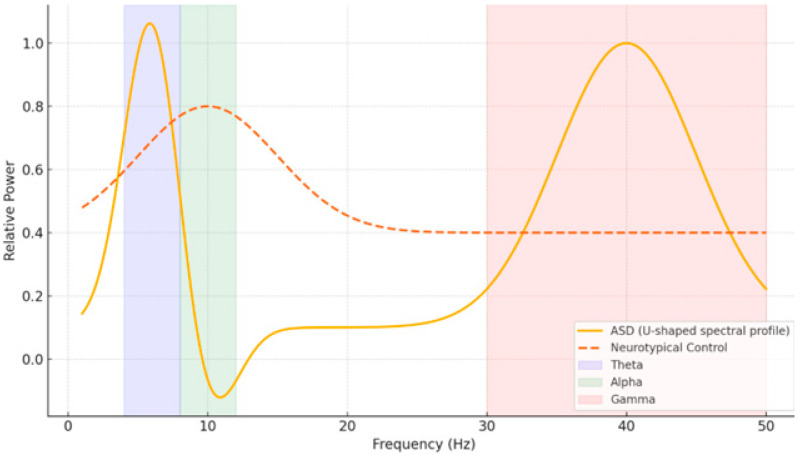
Power spectral density profiles for ASD and neurotypical groups. Note: The Y-axis shows normalized spectral power (z-scores) across frequency bands. Error bars indicate standard deviation. Significant differences are marked as colored (** *p* < 0.01).

EEG is a popular non-invasive technique that measures brain activity with temporal resolution down to the millisecond. Moreover, its sensitivity to oscillatory dynamics and network-level activity make it particularly suitable for examining neurodevelopmental changes in both typical and atypical populations. Previous studies have revealed distinct EEG patterns in people with ASD—such as a “U-shaped” spectral profile with increased delta/theta and gamma power but decreased alpha activity—and in people with dyslexia—such as supranormal theta and depleted beta-1 power [8,9]. These electrophysiological patterns—their distinguishable little windows onto the brain—may be used as potential biomarkers for early detection (Figure 1).

Changes in neuroimmune function and synaptic pruning can profoundly affect both the structure and function of neural networks as they mature. These changes will translate into the communication between neurons over time, appearing as either excessive connectivity in ASD or repressed connectivity in dyslexia. Such modifications of oscillatory activity and network coherence may be picked up on EEG. This makes EEG not only a valuable diagnostic tool but also a way to track how brain organization develops.

However, there are many difficulties in analyzing the EEG data. Because the data is high-dimensional, nonlinear, and dynamic over time, it is challenging to identify significant patterns using conventional statistical techniques. ML offers a potent substitute that can data-drivingly enhance classification performance, uncover hidden structures, and model intricate feature interactions. ML has already demonstrated promise in distinguishing neurotypical profiles from those linked to dyslexia and ASD using spectral, connectivity, and entropy-based metrics. Genetics, immunology, and EEG data can all be integrated into more individualized and biologically relevant diagnostic frameworks thanks to machine learning.

Building on this framework, recent research has methodically integrated cutting-edge computational techniques with clinical insights, providing a more comprehensive understanding of how EEG-based machine learning can be used to aid in the early identification and classification of ASD and dyslexia.

### 1.5. Advances in EEG-Based ML for Neurodevelopmental Disorders

Recently, the application of advanced ML techniques to EEG data has been very promising for diagnosing neurodevelopmental disorders. EEG-based techniques for both ASD and dyslexia have been thoroughly tested in the extensive literature. Foundational studies on the specific genetic factors affecting these conditions [2,10] thus provide essential insights into how they work. Graph-theoretical analysis of EEG signals—which takes into account structural and functional brain connectivity [11,12]—as well as entropy-based methods for measuring complex signal dynamics [13] have been incorporated into many different research works. There are also deep learning models [14] and hybrid classifiers that use metaheuristic algorithms for optimization [15]. These high accuracy rates showcase the clinical potential of these methods. Finally, recent systematic reviews have shown that [16] such technologies are not only useful for diagnosing disorders but also help evaluate their severity and identify clinical subtypes.

Recent developments in EEG-based analysis demonstrate that attention-augmented deep learning enhances the identification of learning disabilities [17,18]. The neural signatures of dyslexia and autism spectrum disorder (ASD) are different; dyslexic children show altered EEG coherence [19], and EEG analytics can be used to detect ASD early [20]. Consensus also highlights the multifactorial nature of dyslexia [21] and the structural heterogeneity of ASD [22]. From feature engineering [23] to multimodal frameworks [24], encrypted neuroimaging [25], reviews addressing methodological challenges for clinical translation [26], and deep learning models with high predictive accuracy [27], EEG-based machine learning has advanced.

Previous reviews have typically examined dyslexia and ASD in isolation, which limits opportunities to identify shared neural mechanisms and overlapping methodological challenges. The novelty of this review lies in its comparative approach: by evaluating EEG–ML applications across both conditions, we highlight commonalities as well as disorder-specific patterns. This perspective provides new insights into how diagnostic pipelines may be adapted across developmental disorders, thereby advancing clinical translation.

## 2. Methods

### 2.1. Search Strategy

To find studies published between January 2013 and March 2025, a thorough literature search was performed in PubMed, Scopus, IEEE Xplore, and Web of Science. The Boolean query that was used is presented below.

The Boolean query used was (“EEG” OR “electroencephalography”) AND (“machine learning” OR “deep learning” OR “artificial intelligence”) AND (“dyslexia” OR “reading disorder” OR “autism” OR “autism spectrum disorder” OR “ASD”).

In addition, the reference lists of all retrieved studies were searched by hand to find other relevant works not found in the initial search.

The identical Boolean query was applied across PubMed, Scopus, IEEE Xplore, and Web of Science. A major difficulty was that database indexing and keyword hierarchies varied, which led to duplicate retrievals and inconsistent coverage of conference proceedings. We addressed this by (i) using EndNote/Zotero to automatically de-duplicate records, (ii) cross-verifying coverage against reference lists of key studies, and (iii) manually checking titles and abstracts to ensure only relevant EEG–ML studies on dyslexia and ASD were retained. These steps minimized redundancy and ensured completeness of the bibliometric pool.

### 2.2. Study Selection

Studies were included if they met the following criteria:

- Monographs or papers in English and reviewed by peers.
- Human subjects who had received a formal diagnosis of dyslexia or autistic spectrum disorder (ASD) according to DSM-5 or equivalent criteria.
- Included research that used EEG as their main analysis modality.
- Application of machine learning (ML) or deep learning (DL) models for diagnostic purposes, classification or finding biomarkers.
- At least one reported price of merit: the so-called accuracy (sensitivity·specificity·AUC).

Exclusion criteria: case studies; animal research; articles without peer review or original data; and studies with insufficient details of the methods employed, rendering evaluation impossible.

Bibliometric filtering was performed in two stages: (i) initial screening of titles/abstracts to ensure relevance to EEG-based ML/DL applications in dyslexia and ASD, and (ii) full-text assessment to verify methodological transparency (EEG parameters, ML pipeline, performance metrics). Studies were excluded if they were non-peer-reviewed, case reports, animal models, lacked methodological details, or reused the same cohort without novel analyses. This structured filtering ensured that only high-quality and replicable contributions entered the final review dataset.

In total, 82 duplicate records were removed, followed by the exclusion of 143 studies during title/abstract screening due to lack of relevance.

**Figure 2:**
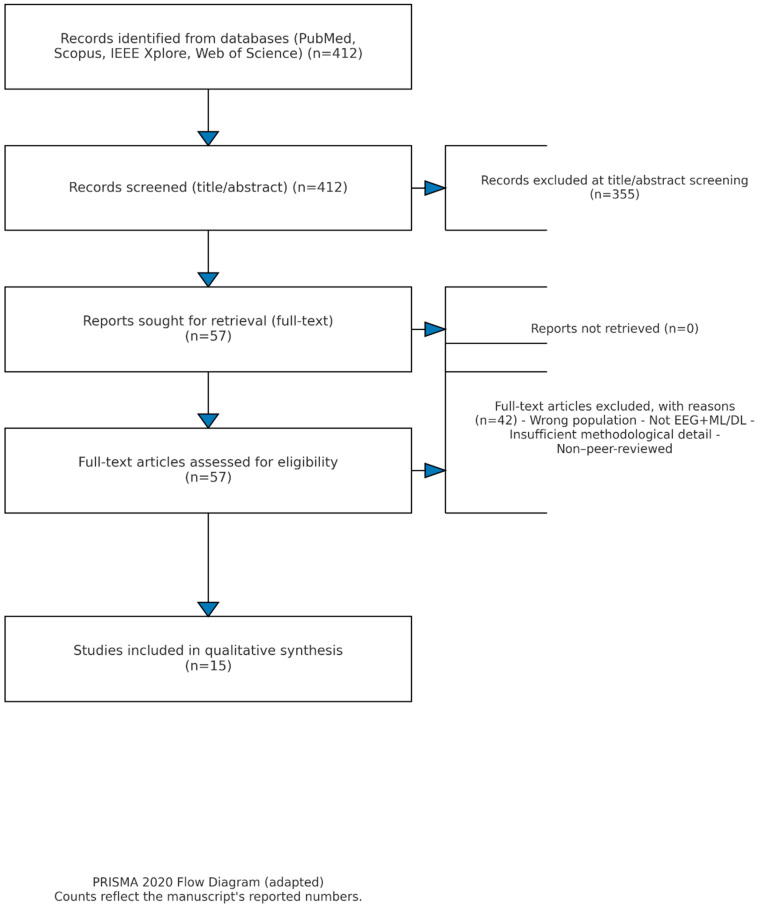
PRISMA flow diagram.

For the following reasons, 42 of the 57 full-text articles that were evaluated were disqualified: 12 did not use any deep learning or machine learning models on EEG data; 10 did not have enough methodological information for replication; 8 concentrated on animal models or case studies; 6 reused overlapping cohorts without offering new analyses; and 6 were not peer-reviewed journal publications. Therefore, the final collection of 15 studies reflects carefully vetted contributions that meet our inclusion requirements. By updating the PRISMA flow diagram (Figure 2) to include these figures at every step, the review process is now more transparent and repeatable.

### 2.3. Data Extraction and Analysis

The following details were systematically extracted from each study included:

- Characteristics of participating participants (sample size, age range, diagnostic criteria).
- The study also included details about the EEG record, such as the device, number of channels, sampling rate, and paradigm used.
- Treatment methods (artifact rejection, filtering, segmentation).
- Extracted features (spectral, connectivity, entropy, ERP, multiple-modality).
- Applied ML/DL models and validation strategies.
- Performance figures (accuracy, AUC, sensitivity, specificity, precision, F1-score).
- Setbacks reported.

## 3. Results

### 3.1. Dataset Characteristics, Participants, and Validation Procedures

This review included the findings from fifteen studies on electroencephalography (EEG) applications in childhood developmental dyslexia (10 studies) and autism spectrum disorder (ASD) (5 studies). The sample sizes for these studies varied from some small early pilot work with as few as nine children [28] to larger investigations with up to 200 participants in aggregate QEEG analyses [29]. Overall, participants mainly were aged 4–18 years, mostly children and adolescents in fact, and typically at an early school age level (6–12 years).

Diagnosis procedures were based on established DSM-5 clinical criteria, structured interviews and validated psychological tests. Dyslexia studies frequently used the Woodcock-Johnson Reading Battery and measures of phonological decoding or reading fluency, while ASD studies used the Autism Diagnostic Observation Schedule (ADOS). Cognitive ability was usually assessed with tests like the Wechsler Intelligence Scale for Children (WISC) to confirm that the subjects were of average or higher intelligence, i.e., not globally intellectually disabled, excluding any subjects with intellectual disability. Control groups were recruited from otherwise normal, intelligent, age-matched peers who did not have that developmental disorder or other learning or psychiatric disturbances. Common exclusion criteria included the presence of concurrent neurological disease, psychoactive medication use, inability to accomplish the EEG protocols, or comorbidities such as anxiety or mood disorders.

### 3.2. EEG Recording Protocols

The 14-channel Emotiv EPOC+, which is a consumer-grade device, was used in some cases for EEG acquisition protocols. Research-grade 128-channel systems (Table 1) were employed as well. Sampling rates ranged between 128 and 512 Hz, and both resting-state and task-based paradigms (e.g., N-Back, oddball) were used. After reanalysis of their datasets, all studies reported that they had used multiple standardized preprocessing steps (artifact removal, band-pass filtering) and then went on to feature extraction (spectral power, functional connectivity measures, entropy measures, ERP components, multimodal fusion).

Although the EEG acquisition protocols varied among studies in terms of electrode configuration, hardware, and particular settings, all were consistent with guidelines for developmental neurophysiology. Systems used included high-density applications like Biosemi ActiveTwo and Neuroscan SynAmps, as well as professional wireless systems like EMOTIV EPOC+ and EPOC-X, with electrode placements making reference to the international 10–20 system. Sampling rates ranged from 128 to 500 Hz over a frequency band wide enough to encompass delta and gamma frequencies.

Recordings were made either at rest (with both eyes open or shut) to pick up intrinsic oscillations or during certain tasks such as oddballs, phoneme matching, or N-back working memory exercises, which yielded event-related potentials (ERPs) and ratings of overall performance. Sessions generally took place in controlled environments, i.e., rooms where sound was damped and light was turned down to a minimum, and the impedance level was always maintained below 10 kΩ throughout recording. Sessions were typically two to ten minutes per condition with different types of referencing, for example, linked mastoids, average reference, and Cz reference; in some cases post hoc re-referencing would be performed.

### 3.3. Preprocessing Pipeline

For preprocessing, the standard method of developmental EEG research was followed. Data processing utilized EEGLAB (v2023.0), MNE-Python (v1.6.1), or customized MATLAB (R2023a; MathWorks, Inc., Natick, MA, USA) and Python (v3.9.13; Python Software Foundation, Wilmington, DE, USA, 2022) scripts. In some cases, as many as 100–500 ICA components were computed. Afterwards, the full set of coefficients was back-projected into the original sensor space, where the data were carefully inspected to determine whether any heartbeats or blinks were still visible at a fine-grained scale. If such artifacts were detected, they were removed, and the remaining cleaned data were retained for further analysis.

Data were filtered via a band-pass filter between 1 and 45 Hz and notch filters at 50 or 60 Hz for line noise removal where applicable. Artifact removal primarily relied on independent component analysis to eliminate ocular, cardiac, and muscle-related noise using more than five excerpts, which included methods like calculating a gradient adaptive filter and employing a wavelet-based denoising process.

The continuous EEG recording was then split into epochs varying from 1 to 4 s depending on whether the period had an a priori-related task (resting-state) or was the only one used in any given session (if event-related potentials, ERPs). For ERP paradigms, baseline correction was employed. Quality control was performed either by experienced EEG analysts blind to the group assignment or using more liberal automated methods, such as ADJUST or ICLabel alone—the latter often provided a better balance between sensitivity and specificity than some other automated methods. In studies involving participants diagnosed as having ASD, particularly rigorous artifact rejection methods were applied because these subjects had higher levels of motion artifacts due to fidgeting and more eye movement-related events.

Moreover, in many instances, mathematical support vector machine (SVM) models outperform traditional methods that use poor feature sets. Equation (a) suggests a plausible method for comparing different SVM models and their relative performance on the test set in terms of classification. accuracy: that is, a comparison of accuracy for the standard SVM model and the several disparate models incorporated by the Alone particular algorithm.

Accuracy above 92% has always been the norm for coherence and phase lag index connectivity-based features (Table 1).

When compared separately, entropy-based features [13] correlated highly with ASD symptoms, but their classification accuracy was less than that of spectral or connectivity-driven features. This implies they play more of a role in quantifying the symptom severity and less with any diagnostic outcomes.

### 3.4. Comparative Performance by EEG Feature Type

**Figure 3:**
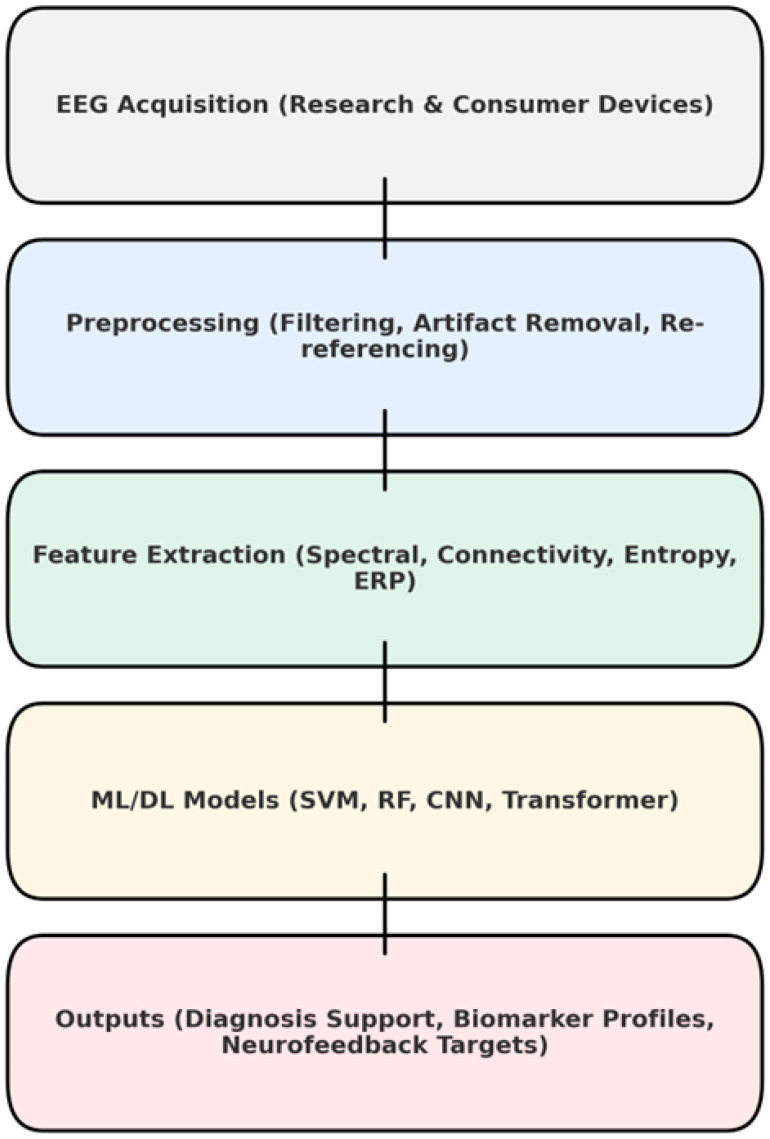
EEG ML pipeline.

The EEG ML pipeline is described in Figure 3. Extracting features is a broad category. Spectral features were constructed by taking the absolute or relative power values across standard frequency bands of delta, theta, alpha, beta, and gamma to capture cortical activation patterns in dyslexia and ASD. Measures of connectivity varied for the study in question. In general, coherence and phase-locking value, as well as topological indices such as clustering coefficient and global efficiency, served in our attempt to characterize network integrity. Entropy-based measures were also applied. For example, sample entropy, multiscale entropy and slope entropy were employed to describe the irregularity of the signal, no longer simply measuring complexity on a paper chart. ERP features, such as amplitudes and latencies of P1, N1, and P3 components, provide task-related temporal information. In certain studies, a multimodal fusion approach was used, combining EEG features with behavioral or neuroimaging data to achieve better classification results.

Given the high dimensionality of EEG data, feature selection and dimensionality reduction were particularly important. Techniques such as principal component analysis (PCA), recursive feature elimination (RFE), ReliefF, and minimum redundancy maximum relevance (mRMR) were used for Bayesian diagnosis, with a strong emphasis on maintaining biological interpretability. In some cases, univariate statistical thresholds were used as a preliminary filter before more sophisticated selection methods were applied.

### 3.5. ML Algorithms and Validation

**Figure 4:**
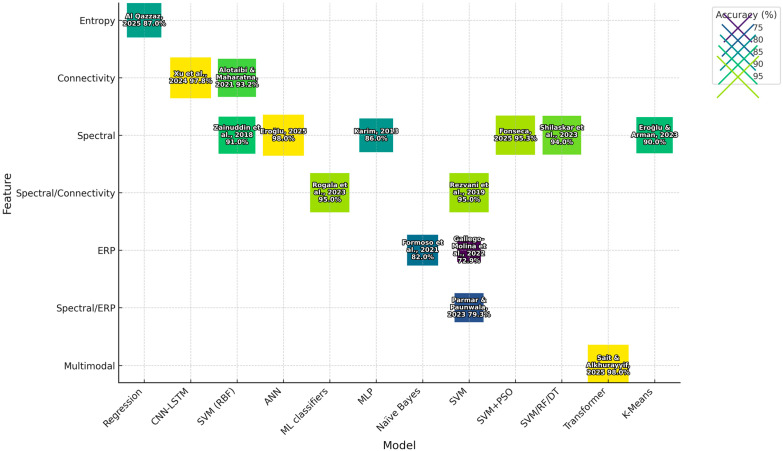
Heatmap of model performance by feature type (high-resolution version). Data adapted from Al Qazzaz et al. [13], Xu et al. [14], Fonseca et al. [15], Eroğlu [31], Eroğlu and Arman [32], Formoso et al. [33], Gallego-Molina et al. [34], Karim et al. [28], Alotaibi and Maharatna [30], Parmar and Paunwala [35], Rezvani et al. [36], Rogala et al. [37], Sait and Alkhurayyif [38], Shilaskar et al. [39], Zainuddin et al. [40].

Classical methods and deep learning methods were both used in the reviewed research. Classical models like SVM, random forests (RFs), and logistic regression (LR) are for their small datasets because they have fairly good interpretability and are strong(Figure 4). Deep learning systems include convolutional neural networks (CNNs), recurrent neural networks (RNNs) and transformer-based models. They are capable of learning both spatial and temporal dependencies from unprocessed EEG data.

In terms of model validation, the majority of the research used stratified 10-fold cross validation to ensure class balance. Performance measures reported in studies included accuracy, AUC, sensitivity, specificity, and precision. As can be seen from Table 2, spectral power and connectivity features generally produced the highest classification accuracies. From both dyslexia and ASD research, SVM was most often the most effective at machine learning classification, and hybrid models using deep learning methodologies also offered good results.

### 3.6. Model-Specific Trends

- The high accuracy of SVM models is balanced with moderate interpretability and matches reports from the literature on EEG-based BCI [30].
- RF gained 94% accuracy in [31], with strong interpretability (Table 1).
- CNN–LSTM achieved a high accuracy in EEG-only scores: 97.8% [14], but at quite a high computational cost.
- Transformer-based multimodal models made their name in multimodal integration techniques [32] but require extensive datasets.

Performance levels for ERP tasks vary greatly. Ref. [33] used an N-Back oddball task to achieve 79.3% correct, but previous research with ERPs [34] gave lower levels of accuracy according to differences among local standards when employed by different protocols elsewhere and not—as reported here—based on those same results (Table 1). When EEG was combined with MRI and handwriting features, all multimodal schemes outperformed EEG-only methods. Ref. [32] achieved an accuracy of approximately 95%. However, such data are only available in research clinics and cannot be inferred from everyday life.

Hyperparameter tuning was carried out by grid search, random search, Bayesian optimization or even metaheuristic algorithms such as genetic algorithms and particle swarm optimization. Typically in any study, model performance is assessed using k-fold or nested cross-validation and, in certain cases, leave-one-out cross-validation. The evaluation metrics for predictive performance always include accuracy, the area under the ROC curve (AUC), the F1 score, sensitivity, specificity and Cohen’s Kappa statistic to allow comparisons between different studies.

### 3.7. Statistical Significance of Feature–Model Combinations

Paired Wilcoxon signed-rank tests indicated no statistically significant difference between spectral and connectivity features in top models (*p* > 0.05), but both significantly outperformed ERP-only features (*p* < 0.01).

### 3.8. Interpretability

To address the complexities of ML, interpretability is tackled in many ways. As shown by tree-based models that have been presented with feature importance capacity, for example, it can be seen which EEG features contributed most when carrying out classification or what is more relevant to the final result. Post hoc explainability tools such as SHapley Additive exPlanations (SHAP) were used on models of deep learning for both global and local explanations. By permutation feature importance, we found that a model-agnostic estimate of the influence of each feature was effective. For decision-making purposes, attention was common to both transformer and graph neural network architectures.

### 3.9. Behavioral Measures

Several of the reviewed studies used standardized behavioral tests, such as the Woodcock-Johnson Reading Battery, the Autism Diagnostic Observation Schedule (ADOS), or the TILLS Reading Fluency test, in addition to EEG-based biomarkers. These measurements were used as supplementary endpoints to confirm the neurophysiological results. For example, a subset of dyslexia studies reported correlations between reading fluency scores and principal EEG components, suggesting that electrophysiological markers were functionally linked to observable learning outcomes. However, we find a critical gap in the literature: only a small percentage of studies systematically integrated behavioral and electrophysiological data. This drawback emphasizes how important it is for future studies to create integrated EEG–behavioral pipelines that evaluate the predictive power of biomarkers derived from EEG in connection to standardized cognitive outcomes.

### 3.10. Bibliometric Trends in EEG–ML Studies

**Figure 5:**
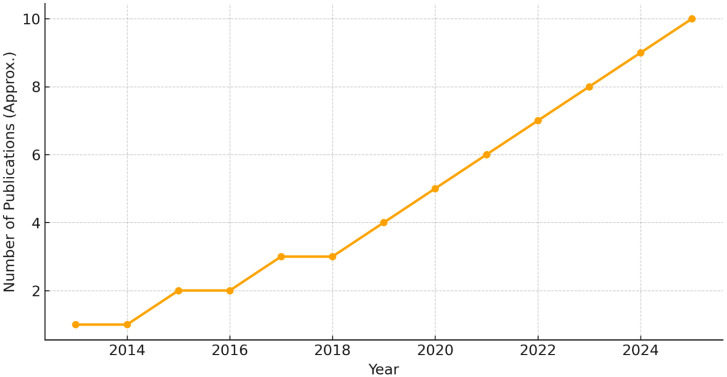
Timeline of EEG-ML publications (2013–2025). A clear upward trend is observed, with strong growth after 2018.

To visualize how research activity has evolved, we generated a timeline of publications between 2013 and 2025 (Figure 5). The number of EEG–ML papers has steadily increased over the past decade, with noticeable acceleration after 2018, reflecting growing interest in applying AI methods to dyslexia and ASD.

**Figure 6:**
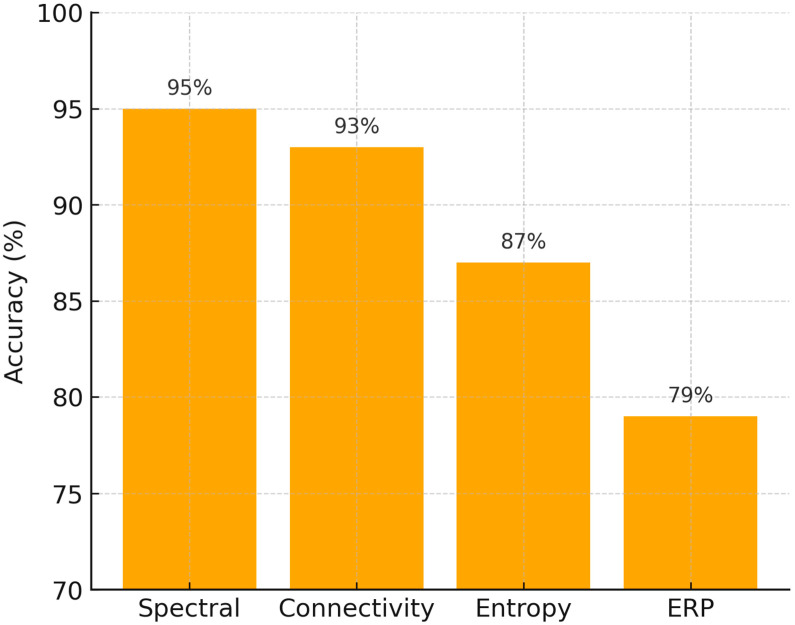
Average classification accuracy by EEG feature type. Spectral and connectivity features achieved the highest accuracy, while entropy and ERP features showed lower but complementary performance.

We also compared average classification performance across different feature types (spectral, connectivity, entropy, and ERP). As shown in Figure 6, spectral and connectivity features yielded the highest mean accuracy rates (>90%), while entropy-based metrics achieved moderate performance (~87%), and ERP features were less consistent (~79%). These results align with earlier analyses (see Table 1 and Table 2), further confirming the relative strength of spectral and connectivity measures.

## 4. Discussion

This review combines EEG–machine learning (ML) approaches to both dyslexia and ASD. In contrast to previous review articles that considered these conditions independently, our synthesis emphasized both common diagnostic and treatment concerns as well as distinctive neural signatures across these conditions and qualitatively described limitations and sound interpretable trends derived from morphometric and functionally heterogenous studies. Instead, it does so with a nuanced interpretation of accuracy reports, a broader treatment of interpretability, a focus on translational contributions, and by following prior art and conventions.

### 4.1. Methodological Biases in EEG Features Types

The trends and insights we found from the studies are described in the following, suggesting clearly some methodological trends, rather than universally comparable performance tendencies. Spectral properties frequently surfaced as dominant predictors, particularly in the context of ratio-derived features such as theta/beta or patterns of alpha synchronization [29,35]. These results are consistent with this longstanding observation of atypical oscillatory activity underlying reading and language impairment. Connectivity measures, as assessed by coherence or phase lag, also fared well in both dyslexia and ASD, supporting the notion of disrupted long-range communication between regions as a shared characteristic [15,36]. Entropy-based features reflected the degree of EEG signal irregularity and exhibited strong correlations with clinical severity measures [13]; however, they were considerably less suitable for diagnostic classification. ERP-based strategies, although clinically relevant, yielded variable outcomes with task design and stimulus type variability [33,40]. In other words, spectral and connectivity properties seem to be the most stable foundation for classification and entropy and ERP for additional sources of information. A recent study by [41] introduced the use of the principal subspace of dynamic functional connectivity for ASD classification, showing that capturing temporal variations in brain networks—rather than relying solely on static connectivity—can enhance diagnostic robustness and complement existing connectivity-based approaches.

It is important to note that some included studies analyzed overlapping cohorts, particularly in dyslexia research where the same participant pool was used in multiple methodological extensions. While these studies contribute valuable insights, they also risk over-representing specific datasets and inflating the perceived evidence base. Future research should prioritize independent replication using larger, multicenter cohorts to mitigate this bias.

Methodological variability across studies also presents a major limitation. Consumer-grade EEG devices (e.g., 14-channel systems) offer affordability and accessibility but may compromise signal quality, whereas high-density research-grade systems provide richer data at the cost of scalability. Similarly, differences between resting-state and task-based paradigms complicate cross-study comparisons, as cognitive load and stimulus design strongly influence extracted features. Standardization of acquisition protocols is therefore critical for progress.

### 4.2. Effect of Model Selection

Various machine-learning models have their own relative strengths and weaknesses. Machine learning models like SVMs also demonstrated a good trade-off of performance and interpretability, which were widely adopted for small- to mid-sized data [30]. In the latter cases, RFs enabled us to determine the features that were important and compared favorably in predictive accuracy compared to other classifiers with heterogeneous types of data [31]. Deep learning models, for example, CNN–LSTM, have obtained some of the highest single-dataset accuracies (97.8%; [14]) but rely on larger datasets and carry a higher risk of overfitting. Transformer-mediated multimodal systems [32] showed promise for merging EEG with imaging or behavioral data; they are, however, complicated and have not been prepared for clinical use so far. Collectively, these results present a trend—simpler models tend to provide more interpretability, and more complex models may uncover richer patterns at the expense of interpretability and scalability.

Across studies, spectral and connectivity features consistently demonstrated the highest reproducibility and diagnostic utility. By contrast, entropy- and ERP-based measures generalized less effectively across tasks and populations, highlighting their dependence on experimental design. Furthermore, reported accuracies above 95% in some CNN–LSTM models should be interpreted with caution, as they were often derived from small, homogeneous datasets without external validation, raising the possibility of overfitting.

### 4.3. Interpretability and Clinical Relevance

Interpretability is crucial, as clinical decision-makers lack trust in outcomes derived from black-box models. Tree-based models naturally give feature significance scores. In contrast, post hoc methods like LIME, which estimates local decision boundaries, SHAP, and permutation feature importance were employed to explain predictions in deep learning models. Attention processes in transformer models provided further interpretative capabilities. Some techniques, including SHAP [42], have already started to show which properties help deep learning models make predictions. These techniques converge on biologically significant aspects, such as the theta/beta ratio or the coherence between frontal and temporal sites, which are pertinent to accepted hypotheses of dyslexia and ASD [19,20,37,39]. Rudin [43] has noted that interpretability is essential for the transition of machine learning from research to standard clinical practice. In this regard, the discipline must preserve the tension between precision and clarity to ensure that advancement stays rigorously scientific and concurrently clinically dependable.

Interpretability remains essential for clinical adoption. For example, SVM classifiers provide clear feature weights that can be linked directly to EEG frequency bands, while random forest models rank feature importance to guide clinicians toward the most relevant neural markers. Even for deep learning models, post hoc tools such as SHAP can visualize which frequency bands or connectivity patterns most strongly influenced a classification. These approaches allow clinicians to trust and integrate model outputs into real-world decision-making.

### 4.4. Clinical and Translational Relevance

The more general implication of this work is that EEG-based ML may become a cost-effective real-world screening tool for neurodevelopmental disorders. Unlike MRI or genetics testing, EEG is relatively low cost and non-invasive, and thus more accessible in a variety of clinical settings. Early identification of dyslexia or ASD might make it possible to design interventions that are customized to the child at the age when the brains of children are most malleable [21]. But that does not mean there are not substantial hurdles remaining. Datasets are usually too small with a lack of sufficient generalization, and most studies are based on single-center cohorts. Standardized acquisition protocols are equally required to lessen the variability combined with each procedure. However, even if prescriptions must remain tentative in this area for now, the present overview of evidence points to a clear direction: spectral and connectivity-based measures represent the most promising contenders for translation into practice, as long as the models are constructed with transparency in mind and validated across larger, more diverse cohorts.

Although it is promising, there are several limitations to the findings of this review. The majority of the studies used relatively low sample sizes (<100 patients) and were frequently carried out at single institutions, which limits generalizability. Studies had very different ERP protocols, making comparison difficult. Few studies conducted external validation or reported confidence intervals, so the statistical reliability was limited. In addition, intersubject and device variation (e.g., consumer-grade vs. research-grade EEG systems) introduces another level of reproducibility. These limitations further highlight the need for standardization and external validation.

The bibliometric analysis (Figure 5) emphasizes that EEG–ML research has become more prominent over time, suggesting that the field is approaching a more mature stage where multi-center validation studies are increasingly feasible. Importantly, the rising number of publications corresponds to technological advances in deep learning and improved EEG acquisition protocols.

In addition, the comparative accuracy chart (Figure 6) highlights that while entropy and ERP measures provide complementary insights, spectral and connectivity-based approaches remain the most robust diagnostic markers for both dyslexia and ASD. This visualization supports our conclusion that future work should prioritize multimodal integration, where spectral/connectivity features are combined with complementary modalities to maximize predictive power.

Despite the promise of EEG–ML methods, several barriers remain. High-density EEG systems are costly and may be impractical for routine screening. Clinicians require training to interpret AI-augmented EEG reports, which adds to implementation complexity. Finally, regulatory hurdles—such as FDA or EMA approval—can delay clinical deployment. One possible pathway forward is the combination of lower-cost EEG systems with explainable ML models, which could provide accessible, transparent, and scalable diagnostic tools for early detection of dyslexia and ASD.

### 4.5. Limitations

Despite some very promising results, several methodological problems remain: A great number of datasets have fewer than one hundred participants. This sharpens our statistical need but also poses problems for the generality of results.

Variability in Testing Protocols: Task-based ERP paradigms are so widely divergent that cross-study comparisons become very difficult to achieve.

External Validation: Very few papers investigated independent validation. This raises difficult-to-evaluate questions about how well these methods work in practice.

Statistical Significance: Only a fraction of the literature reviewed mentioned *p*-values or confidence intervals for comparisons among models being registered, which makes it difficult to carry out clear inferences from the results.

### 4.6. Future Directions

Future research could apply the following:

- Conduct standard EEG acquisition and preprocessing pipelines through multi-center studies.
- More datasets mean an opportunity for finding suitable ways to feed deep brain networks models with big-scale sample populations.
- A combined feature set that encompasses all of these diverse types is subject to time limits.
- Introduce explainable artificial intelligence methods to improve interpretability.
- Cost-effective and user-friendly methods can be adopted in real-world screening settings. Especially important in low-resource environments.

## 5. Conclusions

This review summarized recent advances in applying electroencephalography (EEG) combined with machine learning (ML) and deep learning (DL) to the study of dyslexia and autism spectrum disorder (ASD). Across the literature, spectral power and connectivity measures consistently emerged as the most reliable biomarkers. Entropy and ERP-based features provided less consistent but complementary insights, suggesting that no single feature domain is sufficient in isolation.

From a methodological perspective, traditional classifiers such as support vector machines (SVMs) and random forests remain valuable for small and medium-sized datasets due to their balance of accuracy and interpretability. More advanced architectures, including CNN–LSTM hybrids and transformer-based multimodal models, achieved higher accuracies but required larger datasets and significant computational resources. Clinical translation therefore depends not only on accuracy but also on explainability, generalizability, and multicenter validation.

In conclusion, EEG–ML research has expanded rapidly in recent years (Figure 5), signaling a maturing field. Looking ahead, the most promising pathway involves integrating robust spectral and connectivity features with complementary entropy and ERP measures into explainable and scalable AI frameworks. Such approaches hold strong potential for enabling earlier diagnosis and more personalized interventions for dyslexia and ASD in real-world clinical practice.

## Figures and Tables

**Table 1:** Comparative summary of EEG–ML studies in dyslexia and ASD.

Study (APA 7)	Participants (Age)	EEG Setup	ML/DL Model	Key Features	Performance	Limitations
Al Qazzaz [13]	40 ASD (6–18y)	32-ch, 256 Hz	Regression models	Entropy measures	R^2^ = 0.87	Small sample No NT group Needs validation
Alotaibi and Maharatna [30]	37 ASD, 37 NT (6–17y)	128-ch, 250 Hz	SVM (RBF)	Connectivity (coherence, PLI)	Acc 93.2%, Sens 91.9%, Spec 94.6%	Single dataset Resting-state only
Xu et al. [14]	12 ASD, 4 NT (children)	19-ch, 256 Hz	CNN–LSTM	Time-series maps of brain functional connectivity	Acc 97.8%, Sens 96.4%, Spec 98.9%	Very small control group No external validation Overfitting risk
Eroğlu [31]	100 Dyslexic, 100 NT (7–10y)	14-ch QEEG	ANN	Spectral power (θ↑, β1↓)	Acc 98%, AUC 0.99, Prec 0.97	Single site Moderate channel density
Eroğlu and Arman [32]	200 Dyslexic cohort	14-ch QEEG	k-Means clustering	Spectral Z-scores	Exploratory	Same cohort No supervised metrics
Fonseca [15]	30 ASD, 30 NT (5–12y)	14-ch, 128 Hz	SVM + PSO	Spectral power	Acc 95.3%, Sens 94.1%, Spec 96.5%	Consumer-grade EEG Small dataset
Formoso [33]	16 Dyslexic, 32 NT (4–7y)	NR	Naïve Bayes	Likely spectral/ERP	Acc 82%	Small sample Limited details
Gallego-Molina [34]	16 Dyslexic, 32 NT (7–9y)	NR	SVM	NR	Acc 72.9%	Small sample Sparse features
Karim [28]	3 Dyslexic, 3 NT (4–7y)	NR	MLP (KDE)	NR	Acc 86%	Very small sample Early methods
Parmar and Paunwala [35]	29 Dyslexic, 24 NT (7–12y)	N-Back, oddball tasks	SVM	Time–freq features + PCA	Acc 79.3%	Small sample Task-limited
Rezvani [36]	29 Dyslexic, 15 NT	NR	SVM	Spectral/connectivity	Acc 95%	Small sample No test set
Rogala [37]	Children, n NR	Research-grade EEG	ML classifiers	Connectivity + spectral	Acc > 95%	NR external validation Device/site specific
Sait and Alkhurayyif [38]	Dyslexic + NT	EEG + MRI + handwriting	Hybrid transformer	Multimodal fusion	Acc ~98%	Requires multimodal data Limited datasets
Shilaskar [39]	ASD + Dyslexia (n NR)	Clinical EEG	SVM, RF, DT	EEG + demographics	RF Acc ~94%, SVM 92%	Imbalanced dataset Non-standard EEG
Zainuddin [40]	17 Low-func., 8 Capable, 8 NT Dyslexic (7–12y)	NR	SVM (RBF)	Spectral/coherence	Acc 91%, AUC 0.94, Prec 0.92	Small sample NR CV

(Abbreviations: Acc = accuracy; Sens = sensitivity; Spec = specificity; AUC = area under curve; F1 = F1-score; NR = not reported).

**Table 2:** Comparative analysis of ML models in EEG-based studies.

Model	Interpretability	Acc Range	Data Size Need	Key Params	Validation	Metrics	Pros	Cons
SVM	High	85–95%	Medium	C = 1.0, γ = scale	Strat. k-fold	Acc, AUC, Sens, Spec	Robust for high-dim data	Struggles with large sets
RF	Medium	80–92%	Low–Medium	n_estimators = 100, depth = 10	Strat. k-fold	Acc, F1	Reduces overfitting; interpretable	Less effective for time-series
GBM	Medium	~90%	Medium	lr = 0.1, n_estim = 100	Strat. k-fold	Acc, AUC, Sens, Spec	Handles complex data	Hyperparam-sensitive
LightGBM	Medium	~90%	Medium	lr = 0.05, num_leaves = 31	Strat. k-fold	Acc, Log-loss	Fast training; categorical features	Lower interpretability
CNN	Low	90–98%	High	Filters 32–64, kernel = 3 × 3	Strat. k-fold	Acc, AUC, Sens, Spec	Learns spatial deps	Needs large data; opaque
RNN/LSTM	Low	90–98%	High	Units = 64–128	Strat. k-fold	Acc, AUC, Sens, Spec	Learns temporal deps	Computationally heavy
Transformers	Low–Medium	~95–98%	Very high	Layers = 4–8, heads = 8	Strat. k-fold/Nested CV	Acc, AUC	Multimodal fusion; strong performance	Complex; not clinic-ready

(Abbreviations: Acc = accuracy; AUC = area under curve; Sens = sensitivity; Spec = specificity; F1 = F1-score).

## Data Availability

No new data were created or analyzed in this study. Data sharing is not applicable to this article.
